# Supplementary material for: Extracellular Vesicles Isolated from Plasma of Multiple Myeloma Patients Treated with Daratumumab Express CD38, PD-L1, and the Complement Inhibitory Proteins CD55 and CD59
Source: Cells. 2022 Oct 25;11(21):3365. doi: 10.3390/cells11213365 (PMC9658084; doi:10.3390/cells11213365)
Supplement: Supplementary file 1 [file cells-11-03365-s001.zip › Supplementary figures.pptx]

## Slide 1
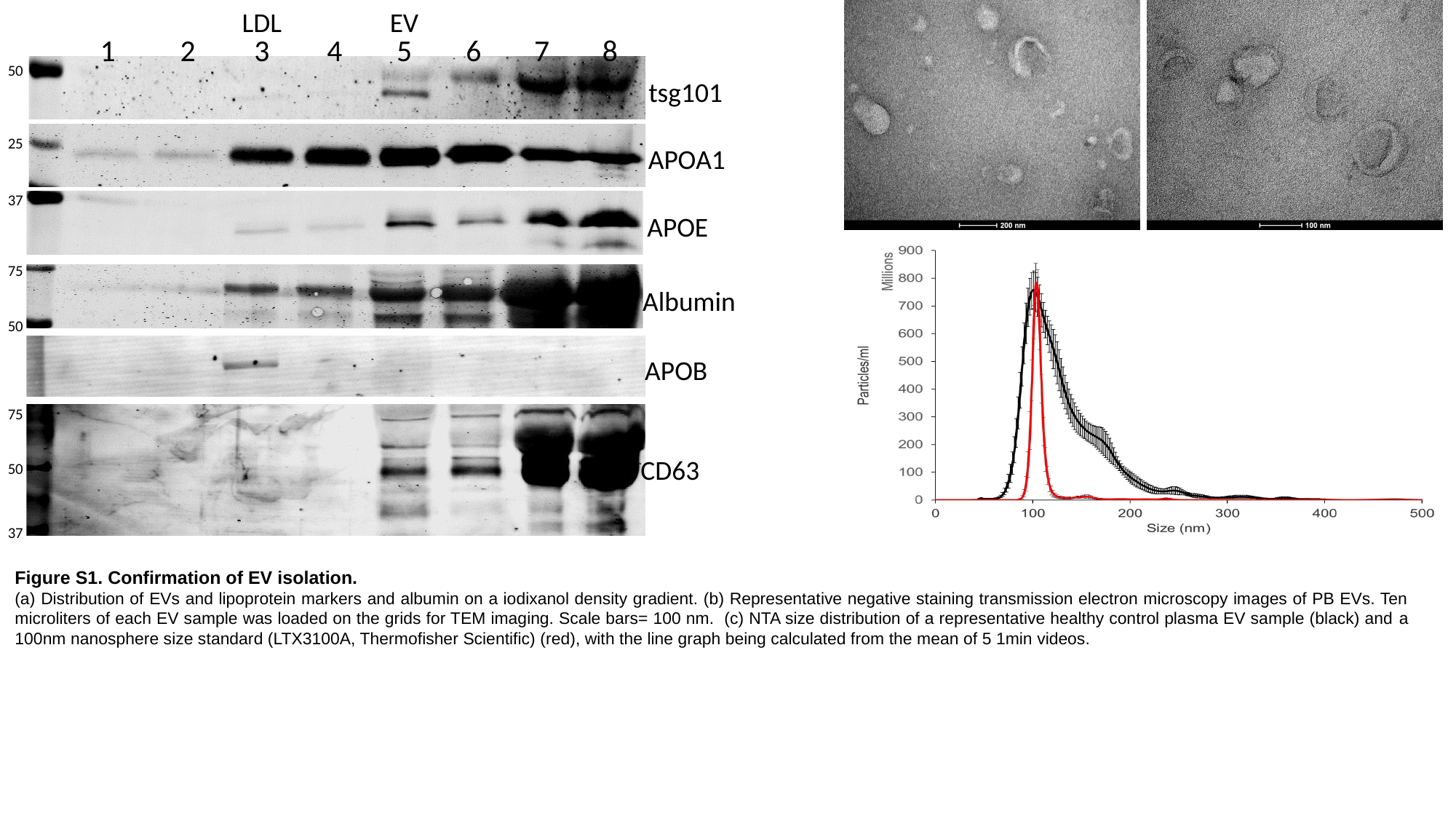

LDL
EV
1
2
3
4
5
6
7
8
50
tsg101
25
APOA1
37
APOE
75
Albumin
50
APOB
75
CD63
50
37
Figure S1. Confirmation of EV isolation.
 Distribution of EVs and lipoprotein markers and albumin on a iodixanol density gradient. (b) Representative negative staining transmission electron microscopy images of PB EVs. Ten microliters of each EV sample was loaded on the grids for TEM imaging. Scale bars= 100 nm. (c) NTA size distribution of a representative healthy control plasma EV sample (black) and a 100nm nanosphere size standard (LTX3100A, Thermofisher Scientific) (red), with the line graph being calculated from the mean of 5 1min videos.

## Slide 2
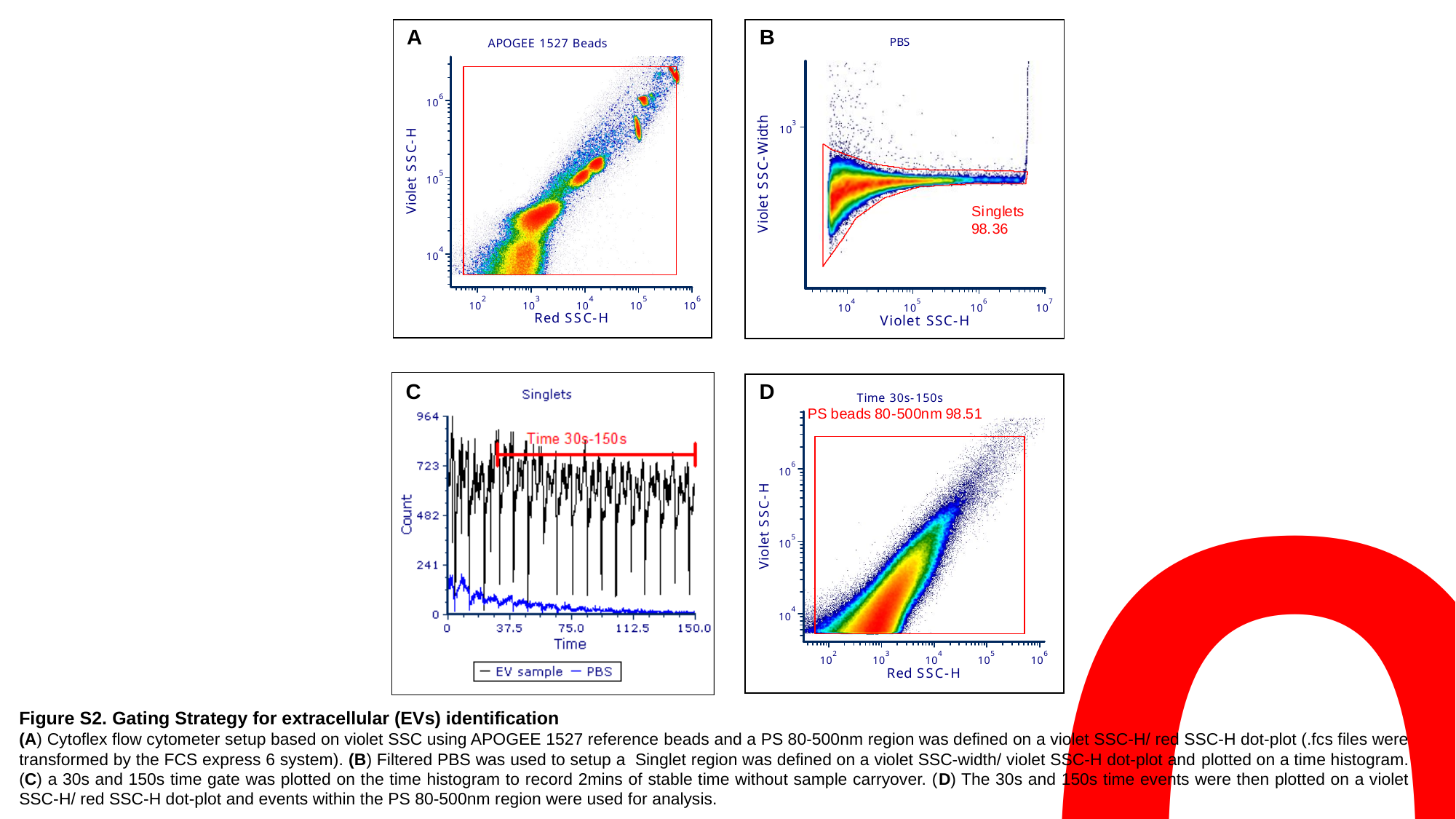

A
B
C
D
Figure S2. Gating Strategy for extracellular (EVs) identification
(A) Cytoflex flow cytometer setup based on violet SSC using APOGEE 1527 reference beads and a PS 80-500nm region was defined on a violet SSC-H/ red SSC-H dot-plot (.fcs files were transformed by the FCS express 6 system). (B) Filtered PBS was used to setup a Singlet region was defined on a violet SSC-width/ violet SSC-H dot-plot and plotted on a time histogram. (C) a 30s and 150s time gate was plotted on the time histogram to record 2mins of stable time without sample carryover. (D) The 30s and 150s time events were then plotted on a violet SSC-H/ red SSC-H dot-plot and events within the PS 80-500nm region were used for analysis.

## Slide 3
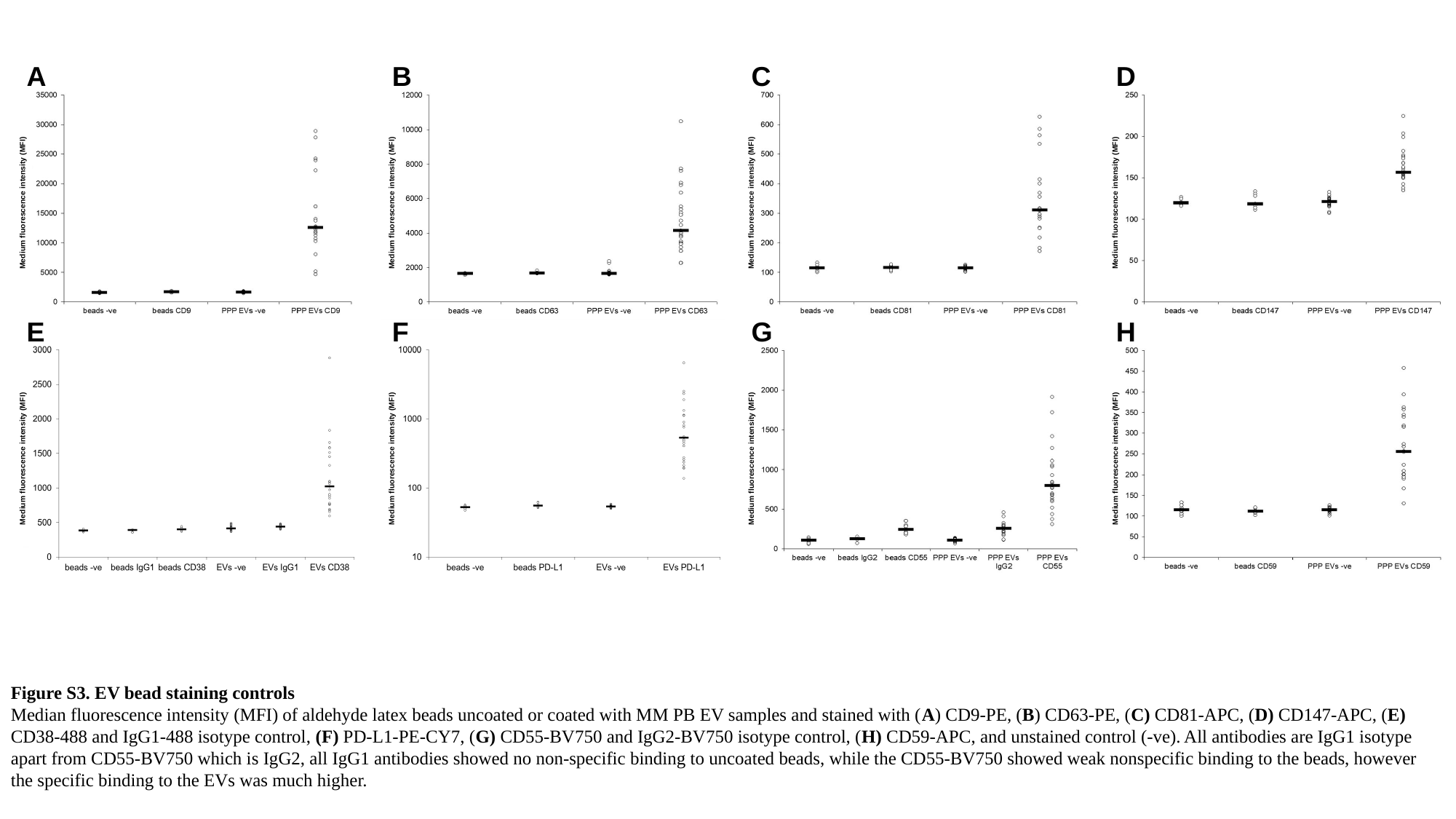

A
B
C
D
Medium fluorescence intensity (MFI)
Medium fluorescence intensity (MFI)
Medium fluorescence intensity (MFI)
Medium fluorescence intensity (MFI)
E
F
G
H
Medium fluorescence intensity (MFI)
Medium fluorescence intensity (MFI)
Medium fluorescence intensity (MFI)
Medium fluorescence intensity (MFI)
Figure S3. EV bead staining controls
Median fluorescence intensity (MFI) of aldehyde latex beads uncoated or coated with MM PB EV samples and stained with (A) CD9-PE, (B) CD63-PE, (C) CD81-APC, (D) CD147-APC, (E) CD38-488 and IgG1-488 isotype control, (F) PD-L1-PE-CY7, (G) CD55-BV750 and IgG2-BV750 isotype control, (H) CD59-APC, and unstained control (-ve). All antibodies are IgG1 isotype apart from CD55-BV750 which is IgG2, all IgG1 antibodies showed no non-specific binding to uncoated beads, while the CD55-BV750 showed weak nonspecific binding to the beads, however the specific binding to the EVs was much higher.

## Slide 4
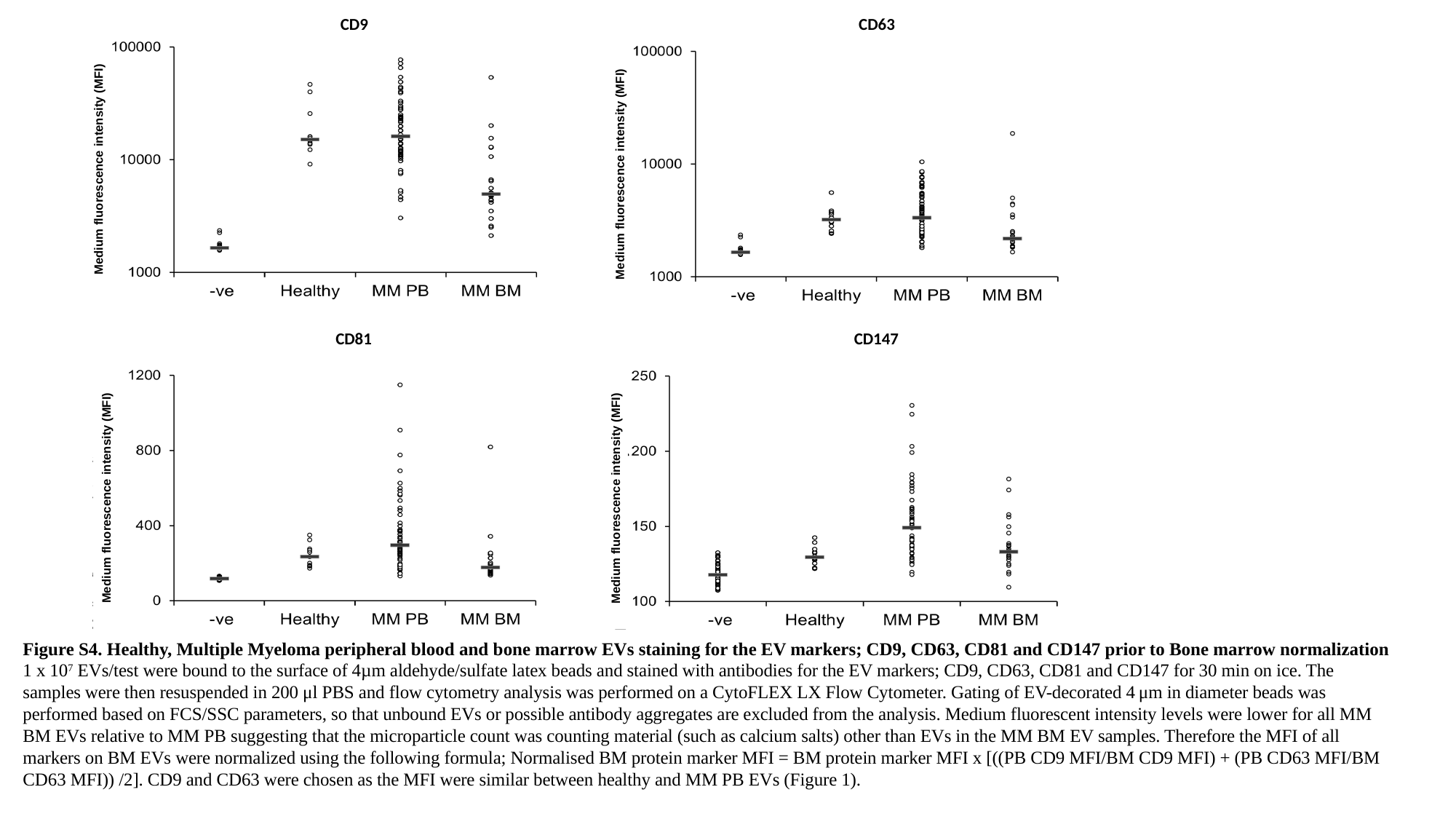

CD9
CD63
Medium fluorescence intensity (MFI)
Medium fluorescence intensity (MFI)
CD81
CD147
Medium fluorescence intensity (MFI)
Medium fluorescence intensity (MFI)
Figure S4. Healthy, Multiple Myeloma peripheral blood and bone marrow EVs staining for the EV markers; CD9, CD63, CD81 and CD147 prior to Bone marrow normalization
1 x 10^7^ EVs/test were bound to the surface of 4µm aldehyde/sulfate latex beads and stained with antibodies for the EV markers; CD9, CD63, CD81 and CD147 for 30 min on ice. The samples were then resuspended in 200 μl PBS and flow cytometry analysis was performed on a CytoFLEX LX Flow Cytometer. Gating of EV-decorated 4 μm in diameter beads was performed based on FCS/SSC parameters, so that unbound EVs or possible antibody aggregates are excluded from the analysis. Medium fluorescent intensity levels were lower for all MM BM EVs relative to MM PB suggesting that the microparticle count was counting material (such as calcium salts) other than EVs in the MM BM EV samples. Therefore the MFI of all markers on BM EVs were normalized using the following formula; Normalised BM protein marker MFI = BM protein marker MFI x [((PB CD9 MFI/BM CD9 MFI) + (PB CD63 MFI/BM CD63 MFI)) /2]. CD9 and CD63 were chosen as the MFI were similar between healthy and MM PB EVs (Figure 1).

## Slide 5
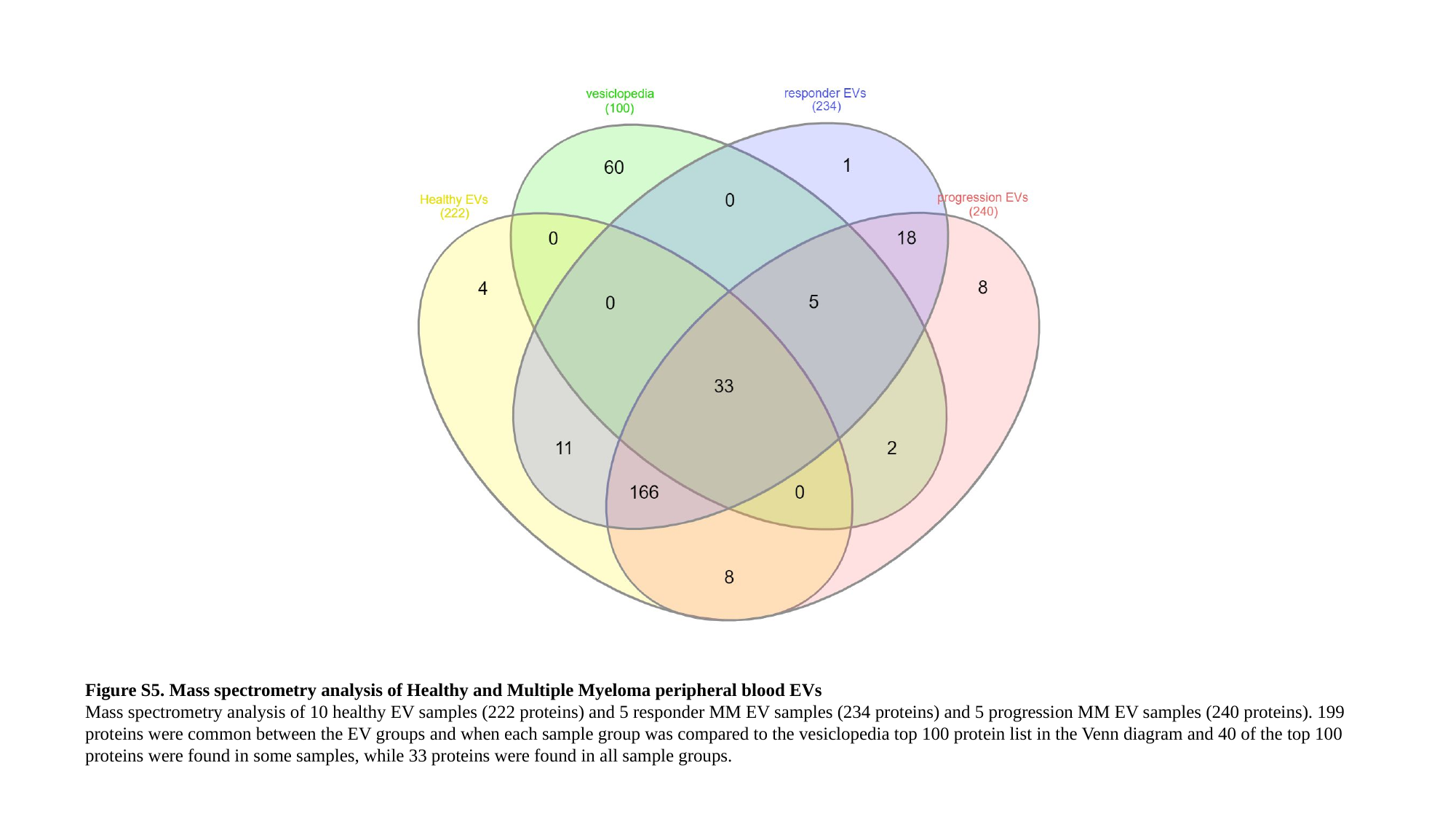

Figure S5. Mass spectrometry analysis of Healthy and Multiple Myeloma peripheral blood EVs
Mass spectrometry analysis of 10 healthy EV samples (222 proteins) and 5 responder MM EV samples (234 proteins) and 5 progression MM EV samples (240 proteins). 199 proteins were common between the EV groups and when each sample group was compared to the vesiclopedia top 100 protein list in the Venn diagram and 40 of the top 100 proteins were found in some samples, while 33 proteins were found in all sample groups.
